# Transformer Feature Enhancement Network with Template Update for Object Tracking

**DOI:** 10.3390/s22145219

**Published:** 2022-07-12

**Authors:** Xiuhua Hu, Huan Liu, Yan Hui, Xi Wu, Jing Zhao

**Affiliations:** 1 School of Computer Science and Engineering, Xi’an Technological University, Xi’an 710021, China; 15938373926@163.com (H.L.); yanxh_xatu@163.com (Y.H.); wuxi786931646@163.com (X.W.); coffeyzj@sina.cn (J.Z.); 2 State and Provincial Joint Engineering Laboratory of Advanced Network, Monitoring and Control, Xi’an 710021, China

**Keywords:** object tracking, transformer architectures, feature enhancement, template update strategy

## Abstract

This paper proposes a tracking method combining feature enhancement and template update, aiming to solve the problems of existing trackers lacking global information attention, weak feature characterization ability, and not being well adapted to the changing appearance of the target. Pre-extracted features are enhanced in context and on channels through a feature enhancement network consisting of channel attention and transformer architectures. The enhanced feature information is input into classification and regression networks to achieve the final target state estimation. At the same time, the template update strategy is introduced to update the sample template judiciously. Experimental results show that the proposed tracking method exhibits good tracking performance on the OTB100, LaSOT, and GOT-10k benchmark datasets.

## 1. Introduction

As one of the challenging and significant research tasks in the field of computer vision, object tracking is the tracking of the position of the target in each frame by using the features of the initial frame in successive video frames in the subsequent frames. Although object tracking algorithms have achieved good results in recent years, the difficulties in the tracking process, such as illumination variations, target appearance variations, occlusion and disappearance, still make object tracking challenging research. We systematically analyzed the literature [1,2] for the current deep learning-based tracking methods, benchmark datasets, and evaluation metrics, which laid the research foundations and research direction for the proposal of this paper.

Currently, most attention is devoted to tracking using the matching ability of the Siamese network. SiamFC [3] uses a Siamese network architecture to extract the features of the upper and lower branch in parallel and determines the target’s location through similarity calculation to balance the accuracy and real-time performance of the tracking problem. Many advanced trackers are algorithmic improvements based on this. SiamBAN [4] adopts a Siamese network structure and anchor-free strategy to remove the pre-defined anchor, thus reducing the model parameters holistically and improving the speed further. ULAST [5] extends the Siamese tracking method by investigating forward and backward video tracking to obtain self-supervised information, which is superior to previous unsupervised methods.

SiamRPN [6] begins with a coarse target localization and then uses the RPN network to process the features to obtain a more accurate target location. To acquire deeper semantic features. SiamRPN++ [7] uses a deep convolutional network and three RPN networks to fuse shallow and deep features, thus increasing the discriminant ability of the network. C-RPN [8] utilizes multiscale features to enhance the feature representation and improve the robustness of the tracking network. Due to the design concept of anchor in RPN, it increases the number of hyperparameters and computational burden to some extent.

DaSiamRPN [9] enriches hard negative sample data by constructing negative samples with semantic meaning. The discriminative learning ability of the network is improved. The idea of SiamDW [10] is like SiamRPN++ in that deeper and wider convolutional networks are introduced into the target tracking algorithm to improve the tracking performance of the algorithm. SiamMask [11] adds the idea of segmentation to the tracker and is a network that can be used to do both video target tracking and video target segmentation.

The attention mechanism can effectively suppress the influence of background interference and target variations on the tracker. To improve the feature representation without introducing too many uncontrollable hyperparameters, researchers have shifted the attention mechanism to the tracking domain. SASiam [12] constructed a two-branch network with semantic and appearance branches and added a channel attention mechanism SENet [13] to the semantic branch to weighted output feature maps. The two branches complement each other to improve feature representation ability. RASNet [14] developed an efficient tracker based on Siamese networks by combining residual attention, channel attention, and general attention. SiamAttn [15] enhanced the features extracted from the benchmark network on three attention modules to improve the robustness of the tracking algorithm. 

Since self-attention can establish long-term dependencies between features. TrTr [16] used a transformer [17] combined with an online update method to improve the performance of the tracking algorithm. TransT [18] employed a transformer to design a tracking algorithm that allows the tracker to obtain better classification and regression results. UTT [19] is a transformer-based tracking algorithm that solves the tracking problem in different scenes using a paradigm. This tracker uses correlation between target features and tracked video frame features for localization of the tracked target. The Stark [20] algorithm models the global spatial–temporal feature relationships using the encoder–decoder transformer structure. The method converts object tracking into a straightforward bounding box prediction problem without employing any suggested or predefined anchors, simplifying the existing tracking pipeline. TransCenter [21] proposes the use of dense pixel-level multiscale queries in a transformer dual decoder network. It can globally and robustly push the heat map at the center of the target, and the robustness of the extra-high tracking algorithm. 

Some classical tracking algorithms consider channel information alone, lacking attention to context information and yielding inadequate feature representation capabilities. Inspired by references [16,17,18,19,20,21] and the advantage that the transformer model can effectively establish associations between feature information, this paper proposes a feature enhancement network with an improved transformer structure.

In the tracking process, the tracking algorithm should be able to adapt to the appearance change of the target in time. TA-Siam [22] designs a template adjustment module that adaptively adjusts the current template using the characteristics of subsequent frames, effectively overcoming the Siamese network model-shifting problem. DSiam [23] proposed a dynamic twin network that incorporates a "generalized morphological learning model" that learns the apparent changes in the target and background suppression online from previous frames.

GradNet [24] proposed a method to apply gradient information to template update, which mainly uses gradient information to update the current template online through forward and back propagation. MemTrack [25] designed a dynamic memory network for object tracking update templates. The network takes control mainly by an external memory block LSTM with attention mechanism and has good adaptability to changes in appearance. This method is too complicated, which increases the computational overhead and affects the real-time performance of the tracking algorithm.

The related research work provides useful ideas for this paper to design feature enhancement networks combining channel attention and transformer models based on the architecture of Siamese networks, as well as being conducive to the paper to design a reasonable and effective model update strategy.

In general, the main contributions of this work can be summarized as:

- In this paper, a feature enhancement module is designed to enhance the spatial–temporal and channel-wise saliency of the features extracted from the benchmark network, which can make the tracker automatically focus on the beneficial feature information and improve the feature characterization ability.
- In this paper, a template update strategy is introduced. By dynamically updating the target template, the impact of target appearance changes on the tracker is better mitigated.
- In this paper, the proposed tracking algorithm achieves state-of-the-art tracking performance on three challenging benchmarks, OTB100, LaSOT, and GOT-10k.

The remaining contents of this paper are organized as follows. In Section 2, we briefly describe the problems to be solved in the paper. In Section 3, our proposed tracking method is explained in detail. In Section 4, our tracking method is evaluated and analyzed on three challenging benchmarks. In Section 5, the whole paper is concluded briefly.

## 2. Problem Description

The fully convolutional Siamese network has two same network branches, which can be used to measure the similarity of two inputs and is suitable for the object tracking task. As a typical, full-convolution Siamese network tracking algorithm, SiamFC, the upper branch is the target template branch, and the lower branch is the search branch. The template image z and the search image x are sent to the upper and lower branch, respectively. The feature vectors $f_{z}$ and $f_{x}$ are taken from the input image by convolutional neural network *φ* (AlexNet). $f_{map}$, the feature response mapping, is obtained after performing the intercorrelation operation (*) on the two feature vectors.

(1) $$f_{z}=\varphi \left(z\right)$$

(2) $$f_{x}=\varphi \left(x\right)$$

where, $f_{z}$ and $f_{x}$ refer to the two feature maps obtained from the template image and the search image through the CNN convolutional network, and *φ*(.) refers to the AlexNet convolutional neural network. 

After obtaining two feature vectors by a fully convolutional Siamese network, we calculate the similarity of the two feature vectors. We use the sliding window to obtain the final response graph. The higher response is considered the location of the target. This method is simple and fast and specifically expressed as:

(3) $$f_{map}=f_{z}*f_{x}+b\;$$

where *b* represents the bias, is the one-dimensional vector, and * represents the convolution operation.

This tracking algorithm uses a shallow network for feature extraction. Due to the small number of convolutional layers, it becomes difficult to extract deep semantic information. Therefore, it cannot take full advantage of the representation capability of a deep convolutional neural network. Meanwhile, the matching template is always the sample template of the initial frame during the tracking process. It cannot accurately adapt to the change of the target in real-time, which is not conducive to the robustness of the tracking algorithm. Moreover, the simple calculation of the distance between feature vectors to determine the similarity of targets has low accuracy.

To achieve better tracking performance, this paper uses deep ResNet50 to extract deeper semantic information. Combining shallow information, channel attention, and transformer architecture, a feature enhancement network to improve feature representation and introduce template updates to adapt to changes in target appearance was proposed. Simultaneously, according to the characteristics of the upper and lower branches of the Siamese network, the tracking task is regarded as two sub-tasks of classification and regression. The classification branch separates the target from the background and the regression branch accurately obtains the bounding box of the target, and locates the tracking target accurately by combining the characteristics of both.

## 3. Transformer Feature Enhancement Network and Template Update for Object Tracking

**Figure 1:**
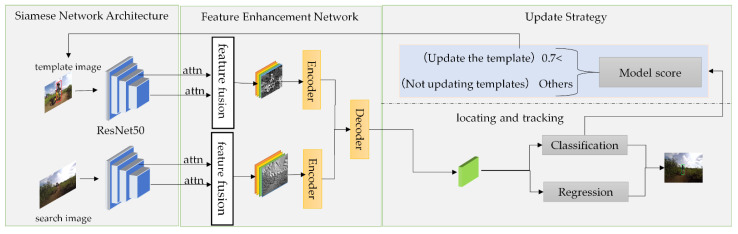
Overall framework of object tracking method based on transformer feature enhancement network with template update.

Based on the SiamFC object tracking algorithm, an object tracking method combining feature enhancement and template update was proposed by dividing the tracking task into two branches, classification, and regression, according to the network structure characteristics of the Siamese network. Specifically, deep semantic features with ResNet50 to extract, and deep semantic features and shallow features for fusion after passing them through channel attention (attn), and the fused features were respectively fused through two encoders to construct the long-term dependence between features. Cross-attention in the transformer decoder was used to exchange information between the features of the two branches, highlight useful global context information and channel information, suppress the interference of similarity targets, and improve the ability of feature representation. In the meantime, a real-time template update strategy was introduced to alleviate the influence of target appearance changes and improve the robustness of the tracker. The overall framework is shown in Figure 1.

### 3.1. Input of The Benchmark Network

ResNet50 network is a benchmark network for feature extraction as used. Compared with the original AlexNet network, the benchmark network can extract deeper semantic information and improve the ability of target judgment and interpretation. For the input to the network, a pair of images selected from the video frames of the training dataset, namely, the template image and search image, feed into the Siamese network architecture and obtain the desired features through the benchmark network.

### 3.2. Feature Enhancement Process

We obtained feature maps with increased representational ability by constructing feature enhancement networks in order to retain more significant target features during tracking and strengthen the correlation between them. The feature enhancement network includes a feature fusion part based on a channel attention mechanism and a long-term dependency establishment part of the transformer.

#### 3.2.1. Feature Fusion Based on Channel Attention Mechanism

Since the convolution operation only takes place in the local space, it is rare to gain enough information to extract the relationships between channels. The channel attention module calculates the weights of each channel of the input image. Then it fuses the shallow and deep features of the channel attention, focusing on the channel containing critical information and enhancing the feature representation ability.

**Figure 2:**
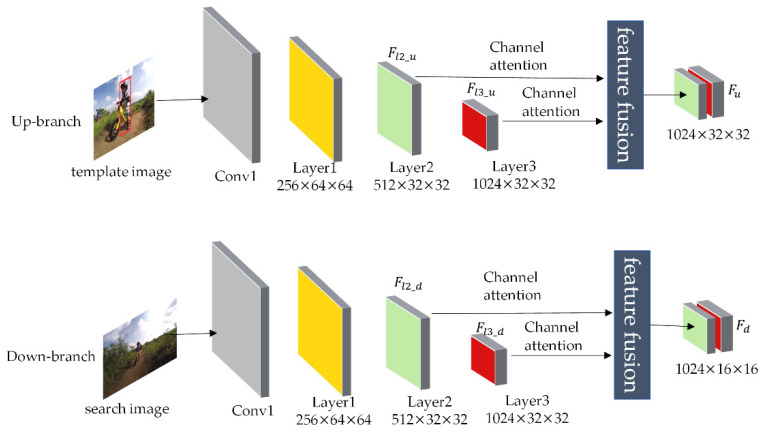
Diagram for fusion of shallow features and deep features.

Template images and search images are passed through the ResNet50 convolutional neural network to obtain the features of the last two stages. Then, they are passed through the channel attention mechanism separately to calculate the channel weight coefficients. The original feature maps are corrected using the resulting channel weight coefficients to obtain the enhanced attention feature maps. Finally, the features of the two stages are fused, where the feature fusion module is up-sampling with bilinear interpolation to unify the features dimension. The fusion process of shallow features and deep features is shown in Figure 2.

**Figure 3:**
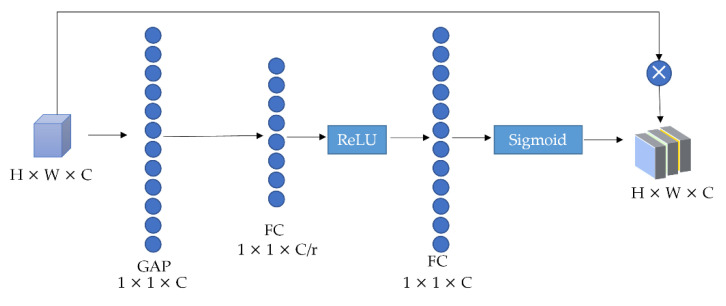
Schematic diagram of channel attention.

The template image and the image go through the four stages of ResNet50. Then the feature vectors $F_{l2\_u}$, $F_{l3\_u}$, $F_{l2\_d}$, and $F_{l3\_d}$ of Layer2 and Layer3 are extracted separately. The critical spatial information of the two-stage feature vectors are enhanced on the channel. The two-stage features are fused using the feature fusion module to obtain the feature vectors $F_{u}$ and $F_{d}$ of the upper and lower branch. The channel attention mechanism corrects the channel features, and the amended features retain the valuable features and eliminate the non-valuable ones. GAP represents global average pooling. A schematic diagram of channel attention is shown in Figure 3.

Firstly, the spatial dimension of the input $F_{l2\_u}$, $F_{l3\_u}$, $F_{l2\_d}$, and $F_{l3\_d}$ feature maps are encoded as a global feature through global average pooling and the input image of size H × W × C to 1 × 1 × C channel descriptors by compression. The specific process of the upper and lower branch features through channel attention is defined as Formulas (4) and (5).

(4) $$S_{lk\_u}=\frac{1}{H\times W}\sum_{i=1}^{H}\sum_{j=1}^{W}\varphi \left(F_{lk\_u}\right)\;\;\;\;k=2,3\;$$

(5) $$S_{lk\_d}=\frac{1}{H\times W}\sum_{i=1}^{H}\sum_{j=1}^{W}\varphi \left(F_{lk\_d}\right)\;\;\;\;k=2,3\;$$

where, $S_{lk\_u}$, $S_{lk\_d}$ denote the global information, H and W denote the height and width of the feature graph, and $\varphi \left(F_{lk\_u}\right)$, $\varphi \left(F_{lk\_d}\right)$ denote the feature vectors of the two stages of the upper and lower branch, and k denotes the index of layer2 and Layer3. The C/r dimensional vector comes from processing the result of the global average pooling through a fully connected layer. Then after performing a ReLU activation function, the C/r dimension vector is transformed back to a C dimensional vector by a fully connected layer. Finally, the final weight matrix is attained by a Sigmoid activation function. The process is defined as Formulas (6) and (7).

(6) $$s_{u}=\sigma \left(W_{2}\delta \left(W_{1}S_{lk\_u}\right)\right)\;\;\;\;k=2,3\;$$

(7) $$s_{d}=\sigma \left(W_{2}\delta \left(W_{1}S_{lk\_d}\right)\right)\;\;\;\;k=2,3\;$$

where, $W_{1}$, $W_{2}$ represent the weights of the two fully connected layers, *δ* and *σ* represent ReLU and Sigmoid activation functions respectively, $s_{u}$ and $s_{d}$ represent the weight matrix finally obtained. Finally, multiply the activation values of each channel learned by the original feature maps.

(8) $$F_{uk}=s_{u}*F_{lk\_u}\;\;\;\;k=2,3\;$$

(9) $$F_{dk}=s_{d}*F_{lk\_d}\;\;\;\;\;k=2,3\;$$

where, $F_{uk}$ and $F_{dk}$ represent channel feature maps combined with weight coefficients; $s_{u}$ and $s_{d}$ represent channel attention weights; $F_{lk\_u}$ and $F_{lk\_d}$ are feature maps of original Layer2 and Layer3. In the whole feature fusion process based on the channel attention mechanism, weight coefficients of each channel are obtained through learning, and corresponding weights are assigned to the original feature graph, thus making the model more discriminating for the features of each channel. After obtaining the two-stage channel feature map, the two fuses receive the feature maps $F_{u}$ and $F_{d}$ with the most representation ability of the upper and lower branch.

#### 3.2.2. Transformer Long-Term Dependency Building Part

The traditional transformer includes an encoder and decoder. The encoder includes two sub-layers, a self-attention module, and forward propagation. The self-attention module serves to learn the internal relationship between features. It can effectively capture the internal correlation between data and features. The decoder consists of three sub-layers, a self-attention module, an interactive attention module, and forward propagation. The self-attention module and forward propagation have the same function as the modules in the encoder. The interactive attention module works to learn the relationship between the original feature and the target feature.

Self-attention gives query(*Q*), key(*K*), and value(*V*) based on the embedded feature vectors and calculates the similarity or association between them based on *Q* and *K*. In this paper, we selected to calculate the similarity using the dot product of two vectors, and the obtained scores as normalized. Then the weighted sum based on the normalized weight coefficients are applied to *V*. The calculation of self-attention can be defined as for Formula (10).

(10) $$self-attn\left(Q,K,V\right)=softmax\left(\frac{QK^{T}}{\sqrt{d_{k}}}\right)V\;$$

where *Q*, *K* and *V* are linear transformations derived from features.

**Figure 4:**
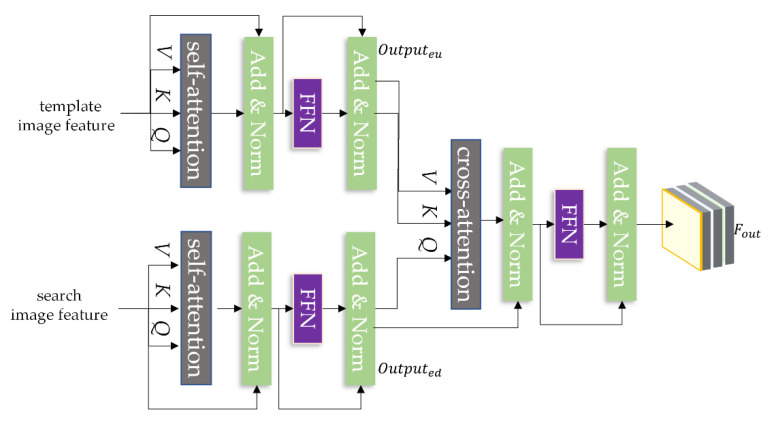
Principle diagram of transformer structure.

To obtain salient features. The internal relationships between the template image feature and the search image feature as learned using the encoder on the upper and lower branch, The learned features are interacted between messages using a decoder without a self-attention mechanism. Among them, FFN is a feed-forward neural network. The principal structure of the transformer designed in this paper is shown in Figure 4.

In this paper, we used a single-headed self-attention mechanism, whose internal operation can be expressed as.

(11) $$Head\left(Q,K,V\right)=Attention\left(QW_{i}^{Q},KW_{i}^{K},VW_{i}^{V}\right)\;$$

where, $W_{i}^{Q}$, $W_{i}^{K}$, $W_{i}^{V}$ respectively represent the weight matrix vectors of *Q*, *K* and *V*, which are the same as *Q*, *K* and *V* from self-attention.

The upper and lower branch encoders receive the channel enhancement feature vectors $F_{u}$ and $F_{d}$. Before receiving the feature, the feature needs a dimensional conversion. The features need to convert into the feature vectors $\tilde{F_{u}}$ and $\tilde{F_{d}}$ required by the encoder. The self-attentiveness of the input template image feature is computed by Formula (10) while adding position encoding at the location of each feature.

(12) $$Output_{eu}=\tilde{F_{u}}+Head\left(\tilde{F_{u}}+P_{z},\tilde{F_{u}}+P_{z},\tilde{F_{u}}\right)\;$$

(13) $$Output_{ed}=\tilde{F_{d}}+Head\left(\tilde{F_{d}}+P_{z},\tilde{F_{d}}+P_{z},\tilde{F_{d}}\right)\;$$

where, $P_{z}$ is the position encoder, $Output_{eu}$ and $Output_{ed}$ represent the output of the upper and lower branch encoder. The decoder is used to interact information on the output of the two branches of the encoder and the feature enhancement network to obtain a high-quality feature vector for classification and regression.

(14) $$F_{out}=Output_{ed}+Head\left(Output_{e_{d}}+P_{q},Output_{eu\_k}+P_{k},Output_{eu\_v}\right)\;$$

where, $Output_{eu\_k}+P_{k}$, $Output_{eu\_v}$ are the *K* and *V* of the encoder branch, the information interaction is carried out in the cross-attention module, and $F_{out}$ is the final output after the information interaction of the upper and lower branch features by the decoder, which is used for the subsequent positioning and tracking. The bounding box $B^{*}$ of the target is determined by classification and regression networks.

### 3.3. Design of Update Strategy

**Figure 5:**
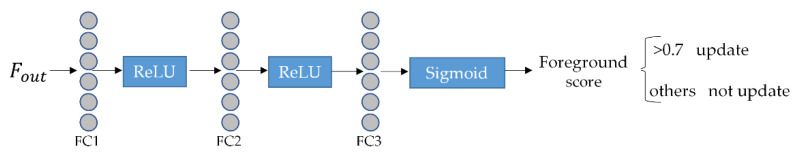
Principle diagram of template update strategy based on classification branch.

In the tracking process, if the target disappears or is blocked, it is not recommended to update the template. In this case, judgment needs to be made on the obtained template to avoid updates leading to a deterioration of the tracking effect. In this paper, target confidence scores judgment by adding a target confidence score at the position of the classification branch. The classification branch has 1024 vectors of length 2, representing the foreground and background scores. The foreground score with the maximal confidence score is extracted and compared with the set threshold. Scores that exceed the set threshold α (>0.7) update their images to the position of the initial frame. This method can make full use of the temporal contextual information of the tracking process and alleviate the problem of changing target appearance. Where FC1, FC2 and FC3 denote fully connected layers, the classification branch-based template update strategy is shown in Figure 5.

### 3.4. Algorithm Implementation

During the implementation of the whole tracking algorithm, offline training was carried out using the publicly available datasets LaSOT [26], GOT-10k [27], and COCO [28] to obtain the final network model. The target state of the template image was pre-set. The status of the search image was determined by network model tracking prediction. The shallow and deep features performed fusion after passing through the channel attention module. The fused upper and lower branch features were dimensionally adjusted and input to the two encoders to build the internal connection between the features. Finally, the output of the two encoders was used for information interaction with the decoder to achieve feature enhancement. The update strategy can effectively alleviate the influence of the change in target appearance. Updating the initial template from subsequent frames can enrich the time context information in the tracking process and make the model more robust. Finally, the location of the target was determined by classification and regression. The specific implementation process is as follows (Algorithm 1).

**Algorithm 1** Procedure of the proposed method
$F_{l2\_u}$, $F_{l3\_u}$ ←$\;\varphi \left(z\right)$; # Feed the template into CNN and the feature vectors of the last two stages are taken outrepeat

$F_{l2\_d}$ $,\;F_{l3\_d}$ $\;\leftarrow \;\varphi \left(x\right);$

$s_{u}$, $s_{d}\;\leftarrow \;\mathrm{attn}\;(F_{l2\_u}$$,\;F_{l3\_u}$$,\;F_{l2\_d}$$,\;F_{l3\_d});$ # Feed the features to the channel attention network attn, get the weight matrix $s_{u}$, $,\;s_{d}$$F_{u}$$(F_{d}$$)\;\leftarrow \;s_{u}$$(s_{d}$$)*F_{lk\_u}$$(F_{lk\_d})\;k=2,3;$ # Fuse the weight matrix and the original feature vector. Get the feature map $,\;F_{u}$$,\;F_{d}$$Output_{eu}$$(Output_{ed}$$)\;\leftarrow \;\tilde{F_{u}}$$(\tilde{F_{d}}$$)+\mathrm{Encoder}\;(\tilde{F_{u}}$$(\tilde{F_{d}}$$));\;\#\;\mathrm{Feature}\;\mathrm{map}\;F_{u}$$,\;F_{d}$$\;\mathrm{is}\;\mathrm{processed}\;\mathrm{into}\;\tilde{F_{u}}$$,\;\tilde{F_{d}}$ and input to Encoder$F_{out}$$\;\leftarrow \;\mathrm{Decoder}\;(Output_{eu}$$,\;Output_{ed});$ # Fuse the Encoder information in the decoder. Get the final feature map $F_{out}$$B^{*}$$\;\leftarrow \;\mathrm{Classification}\;(F_{out}$$)\;\mathrm{and}\;\mathrm{Regression}\;(F_{out});$ # Determining the location of targets through classification and regression networks$\mathrm{Foreground}\;\mathrm{Score}\;\leftarrow \;\mathrm{Classification}\;(F_{out});$ # Foreground score is greater than 0.7 and the template is updateduntil end of sequence


## 4. Experimental Results and Analysis

In order to validate the proposed tracking method, the model was initialized using the parameters pre-trained on ImageNet [29]. LaSOT [26], GOT-10k [27], and COCO [28] datasets were used for offline training of the whole network. During training, the template image was 127 × 127, the search image was 256 × 256, and batch size was 64, optimized using the AdamW [30] optimizer. The learning rate of the benchmark network was set as $10^{-5}$, the learning rate of other modules was set as $10^{-4}$, weight decay and momentum were $10^{-4}$ and 0.9, $L_{1}$ loss and $L_{Giou}$ loss weight were 5 and 2, respectively. 

### 4.1. Quantitative Analysis

To quantitatively analyze the overall performance of the tracker under the interference of complex background and other factors, the proposed method was compared with typical tracking algorithms on OTB100 [31], GOT-10k [27], and LaSOT [26] test datasets.

#### 4.1.1. Comparative Analysis with Typical Tracking Algorithms on OTB100 Dataset

The OTB100 dataset contains 11 tracking challenge factors, 98 videos, and 100 test scenarios, enabling fair and accurate evaluation and comparison of various tracking algorithms. In this paper, the compared method was evaluated with the one-pass evaluation (OPE), and two main metrics of precision plots and success rate plots were adopted to evaluate the performance.

**Figure 6:**
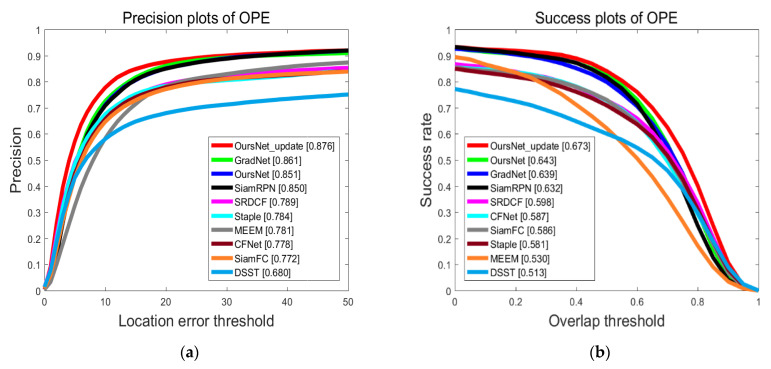
Performance evaluation results of different algorithms on OTB100 dataset. (**a**) Precision plots; (**b**) success plots.

The trackers compared on the OTB100 dataset included SiamFC [3], SiamRPN [6], CFNet [32], GradNet [24], SRDCF [33], Staple [34], DSST [35], and MEEM [36]. Figure 6 shows the precision and success rate plots for eight state-of-the-art (SOTA) trackers on the OTB100 dataset.

As can be seen from Figure 6, the performance of the proposed tracking algorithm was significantly better than that of SiamFC and other algorithms. Compared with SiamFC, the precision and success rate of the proposed method were improved by 10.4% and 8.7%, respectively, due to the addition of the transformer feature enhancement network and template update strategy to the base network SiamFC, indicating the effectiveness of the proposed method. 

CFNet combines correlation filtering with CNN, using a correlation filtering layer to implement online update function, which allows the network to perform well with shallow features. The performance was still not as good as the state-of-the-art trackers due to the boundary effects that CF has difficulty handling.

SiamRPN algorithm uses shallow AlexNet for feature extraction, but it cannot extract deep semantic features and ignores the context information. The tracking algorithm proposed in this paper uses ResNet50 to extract deep semantic features. In addition, the proposed feature enhancement network could effectively improve the feature representation ability. Compared with SiamRPN, the precision and success rate of the proposed method in this paper are improved. 

The accuracy of the GradNet algorithm exceeded that of the tracking algorithm proposed in this paper. However, the success rate was slightly lower than the algorithm proposed. It indicates that the proposed tracking algorithm could adapt the bounding box to the target size well when tracking the target. 

Experimental results show that this method is superior to some classical tracking algorithms in precision and success rate.

**Figure 7:**
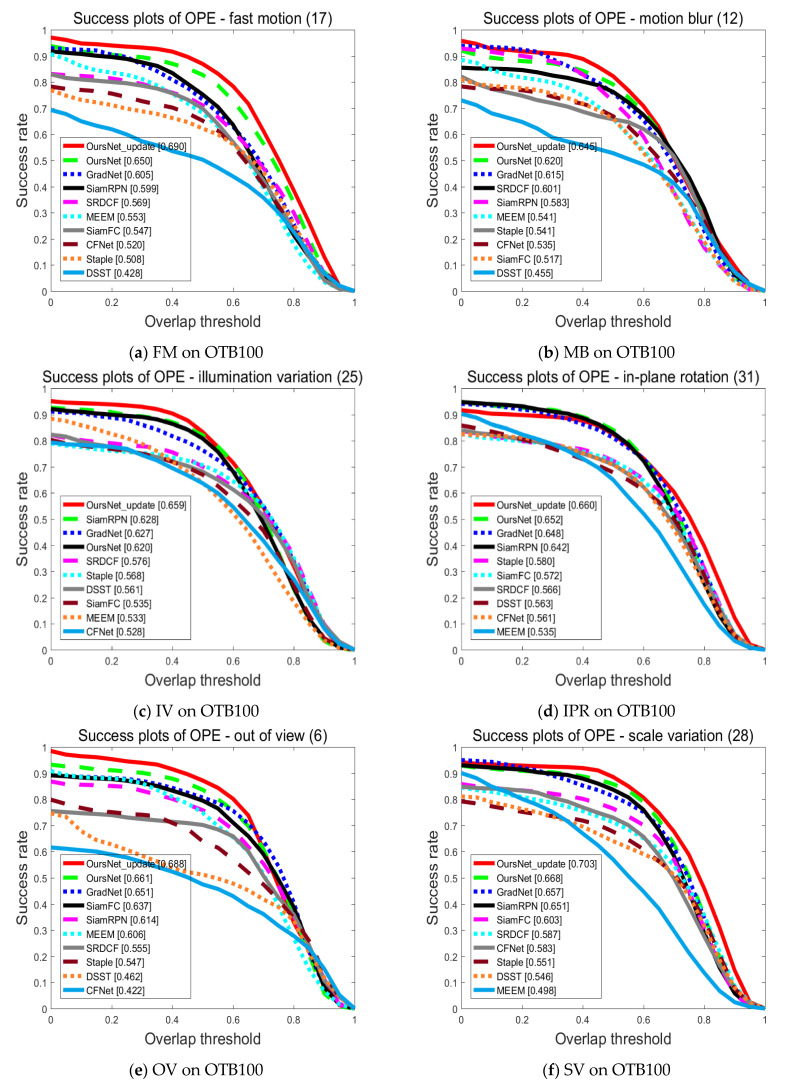

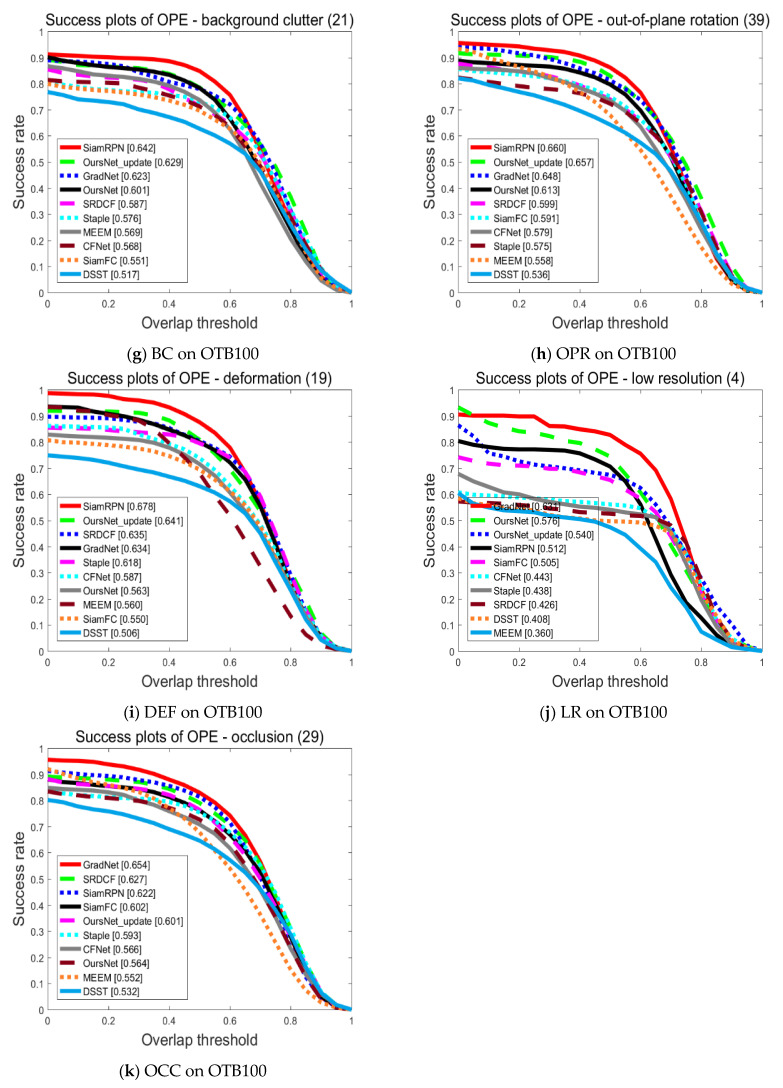
Performance evaluation results of different algorithms on OTB100 dataset for various attributes.

Figure 7a–k provides the detailed results of the success rate on OTB100 for eleven attributes, including fast motion (FM), background clutter (BC), motion blur (MB), deformation (DEF), illumination variation (IV), in-plane rotation (IPR), low resolution (LR), occlusion (OCC), out of view (OV), scale variation (SV), out-plane rotation (OPR). For example, in the case of fast motion, a high adaptive capability of the tracking network becomes necessary. The transformer in the feature enhancement network proposed in this paper could effectively build the long-term dependence relationship of features and accurately obtain feature information in the case of fast movement. In the motion blur case, the tracking network needs to be capable of accurate target tracking in the low-resolution video frame. The transformer feature enhancement network proposed in this paper can make up for the influence of motion blur on the tracker and improve the performance of the tracker.

Further to Figure 7a–f, in this paper, the proposed tracking algorithm was achieved by introducing the transformer feature enhancement network and template update strategy onto the basic network SiamFC, thus, making the tracker perform better on illumination changes, motion blur, fast motion, and target disappearance challenge factors. The robustness of the tracking algorithm was further improved. The transformer feature enhancement network enabled the tracking algorithm to obtain the most representative feature vector, and the template update strategy enabled the tracking algorithm to adapt to the appearance change of the target well.

From Figure 7g–i, the SiamRPN had good tracking performance under the factors of background interference and out-of-plane rotation. However, due to the shallow benchmark network used, the semantic features were difficult to acquire. In addition, in the tracking process, it was difficult to adapt to the situations when the target was moving fast or the appearance changed only depending on the feature information of the initial frame. At the same time, due to the design of the anchor frame, too many super parameters were introduced, so the speed was relatively slow.

As observed in Figure 7j,k GradNet outperformed other trackers due to the inclusion of gradient information and online template updates, under the low resolution and occlusion challenging factors of the OTB100 dataset. Because GradNet mainly solves the problem of updating templates online, it does not deal with the ability to represent features. Therefore, the performance was slightly lower than the tracking algorithm proposed in this paper under the conditions of motion blur and illumination variation.

#### 4.1.2. Comparative Analysis with Typical Tracking Algorithms on LaSOT Dataset

The LaSOT dataset contains 1400 videos with an average of 2512 frames per sequence. Each frame is carefully inspected and manually labeled, with results visually verified and corrected when needed, representing by far the largest single-target tracking dataset with dense annotation for depth tracking training and realistic evaluation of tracking algorithms. We used two evaluation metrics, the normalized precision plots $P_{\mathrm{Norm}}$ and the success plot AUC, for performance evaluation. AUC represents area under curve, $P_{\mathrm{Norm}}$ denotes normalized precision rate.

**Figure 8:**
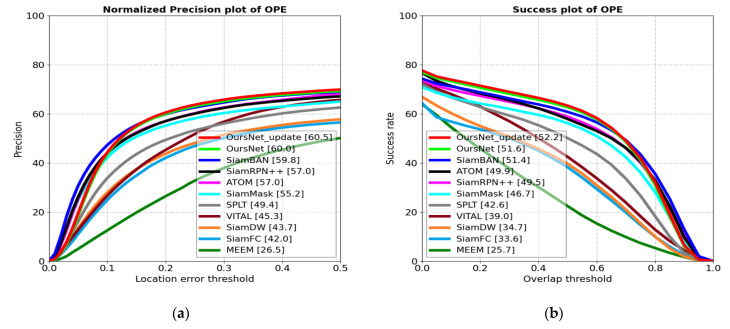
Performance evaluation results of different algorithms on LaSOT dataset. (**a**) Normalized precision plots; (**b**) success plots.

The trackers compared on the LaSOT dataset include SiamBAN [4], SiamRPN++ [7], ATOM [37], TrTr-online [16], TrTr-offline [16], SiamFC [3], SiamMask [11], SiamDW [10], VITAL [38], SPLT [39], MEEM [36], UpdateNet [40], DSiam [23], and ECO [41]. Figure 8 shows the normalized precision and success rate plots for nine SOTA trackers on the LaSOT dataset.

We can see this in Figure 8. The proposed tracker achieved better performance in the long-term tracking dataset LaSOT attributed to the introduction of the proposed feature enhancement network and template update strategy. The tracking algorithm improved accuracy by 18.5% and the success rate by 18.6%, compared to the base SiamFC network. 

The biggest contribution of SiamRPN++ is to change the network structure of twin trackers, using a deeper network instead of a shallow network. The main drawback is that there is no template update, and it is not possible to change the template adaptively when the target appearance changes. Furthermore, the custom anchor frame setting introduces a fuzzy similarity score, which severely hinders robustness and leads to a lower performance compared to advanced trackers.

SiamBAN is an anchor-free based object tracking algorithm that may not contain sufficient target information for accurate regression. Moreover, it lacks the flexibility to handle target changes because it does not consider the internal relationship between features and uses only the initial frame as a template. Therefore, the results are not very effective on the long-time dataset LaSOT.

SiamMask adds a segmentation branch alongside the classification branch and the regression branch, which causes the whole network to be difficult to train. Although ResNet50 is used as the network for extracting features, there is still much room for improvement in robustness.

The SiamDW tracking algorithm ignores changes in the target motion state, resulting in tracking failure when challenging factors such as rapid target motion and occlusion occur.

In this paper, the proposed feature enhancement network not only considered the key information on the channel, but also used the self-attention mechanism to establish long-term dependencies between features and improve the correlation of features, and the template update strategy could effectively adapt to the changes of target appearance. Therefore, the performance was slightly higher on long-time datasets LaSOT compared to trackers such as SiamBAN, SiamRPN++, SiamMask, and SiamDW.

Specifically, the compared performance evaluation results of the proposed method and typical template update trackers on LaSOT dataset are given in Table 1.

The method used only the feature enhancement network proposed in this paper before introducing the template update strategy. The performance was over 5.3% compared to the TrTr-offline tracking algorithm that also uses the transformer architecture. This result shows that the features in the upper and lower branch were fused separately after passing through the channel attention. Then, the association between the features within each branch was performed by the encoder. Finally, the information from the upper and lower branch interacted through the decoder. The performance was improved compared to the decoder process where the upper branch is passed through one encoder and then inputs it to the lower branch.

The performance of TrTr-online tracking algorithm exceeded the tracking algorithm proposed in this paper. It is a combination of classification plus an online update method, which is slower. However, the tracking algorithm in this paper used a simpler linear update mechanism. The real-time performance of the tracking algorithm was preserved with a slight performance improvement.

UpdateNet-DaSiamPRN and UpdateNet-SiamFC were tested by using UpdateNet network on the original DaSiamRPN and the base network SiamFC. However, the performance was lower compared with the tracking algorithm proposed in this paper that combines the transformer and simple update mechanism strategies. This reflects the effectiveness of the tracker proposed in this paper.

The DSiam tracking algorithm learns an online update transformation in two separate branches to update the tracking model through a fast transformation module. However, the tracking method lacks the detection of tracking failures, resulting in low performance.

The ECO tracking algorithm updates the model every few frames to avoid model overfitting. However, its adaptability is reduced due to the underutilization of information during model updates.

As can be seen from Table 1, the tracking algorithm proposed in this paper significantly outperformed typical tracking algorithms such as DSiam and ECO in terms of performance.

#### 4.1.3. Comparative Analysis with Typical Tracking Algorithms on GOT-10k Dataset

**Figure 9:**
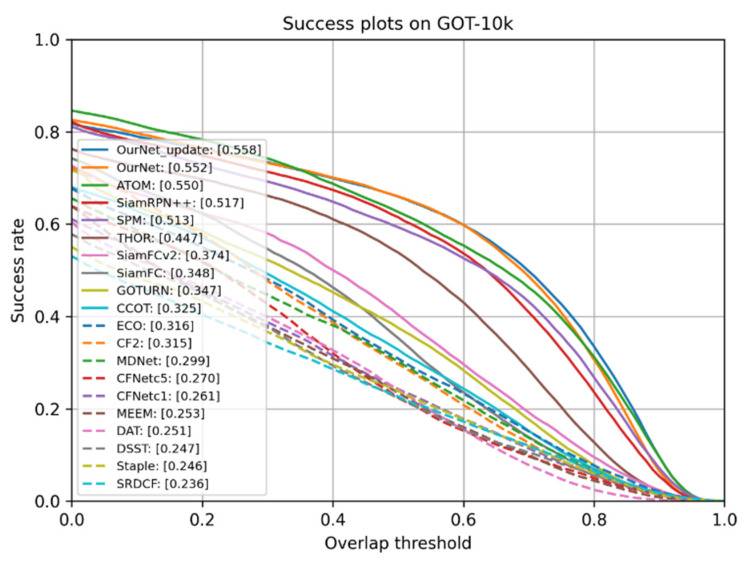
Comparison results of the GOT-10k benchmark.

The GOT-10k dataset contains more than 10,000 real-world video clips and more than 560 categories, with more than 1.5 million manually marked frames, featuring rich scenes and difficult algorithm challenges. Average overlap (AO) and success rate (SR) are two evaluation metrics for the GOT-10k dataset, and ${\mathrm{SR}}_{0.5}$ and ${\mathrm{SR}}_{0.75}$ are two thresholds for the success rate. Therefore, the tracking algorithm proposed in this paper was tested on this dataset. The output results are uploaded to the official website to obtain the performance results of the test. The fairness and effectiveness of the tracking algorithm in this paper as demonstrated. Figure 9 shows the success rate plots for 18 SOTA trackers. It can be observed from the figure that the tracking algorithm proposed in this paper performed significantly better than the others on the GOT-10k dataset.

Table 2 shows the performance details of the tracking algorithm proposed in this paper compared with typical tracking algorithms on the GOT-10k test dataset. The tracking algorithms compared include, SiamFC [3], SiamRPN [6], SiamRPN++ [7], MDNet [42], ATOM [37], ECO [41], THOR [43], SPM [44], GOTURN [45], Staple [34], and SRDCF [33].

ATOM refines multiple initial bounding boxes iteratively, which provides a significant improvement in tracking accuracy. However, the performance was slightly lower than the tracking algorithm proposed in this paper. Meanwhile, the method of target estimation in ATOM not only brings a heavy computational burden but also presents many additional hyperparameters that require careful tuning.

The SPM tracker distinguishes targets from backgrounds by introducing additional relational networks. However, the use of a matching mechanism only and the lack of online updates severely limit the performance of this tracker in dealing with similarity interferers.

THOR is a tracking algorithm based on dynamically updated target templates. The tracker uses greedy search in a local search window, resulting in a less than friendly performance in challenging benchmark tests.

GOTURN uses a fully connected network to estimate corner coordinates directly, while objects in the background also have corner points. Furthermore, the relationship between the template and the test image is not effectively probing. Therefore, it is not easy to distinguish them from the corner points of the tracked target. To some extent, it affects the performance of the tracker.

The MDNet tracking algorithm does not incorporate any feature enhancement operation during feature extraction. Moreover, fine-tuning online makes it slower and requires time and effort to fine-tune. Therefore, the robustness of the tracking performance needs to be improved.

As can be seen in Table 2, the proposed tracking algorithm showed superior performance results in AO, ${\mathrm{SR}}_{0.5}$, and ${\mathrm{SR}}_{0.75}$ metrics. Compared to the base network SiamFC, AO, ${\mathrm{SR}}_{0.5}$ and ${\mathrm{SR}}_{0.75}$ were improved by 21, 30.7 and 32.9%, respectively. Compared with SiamRPN++, which introduces the RPN network, AO, ${\mathrm{SR}}_{0.5}$, and ${\mathrm{SR}}_{0.75}$ were improved by 4.1, 4.4 and 10.2%, respectively. The proposed tracking algorithm also outperformed the THOR and ECO tracking algorithms that use the same dynamic update template.

### 4.2. Qualitative Analysis

**Figure 10:**
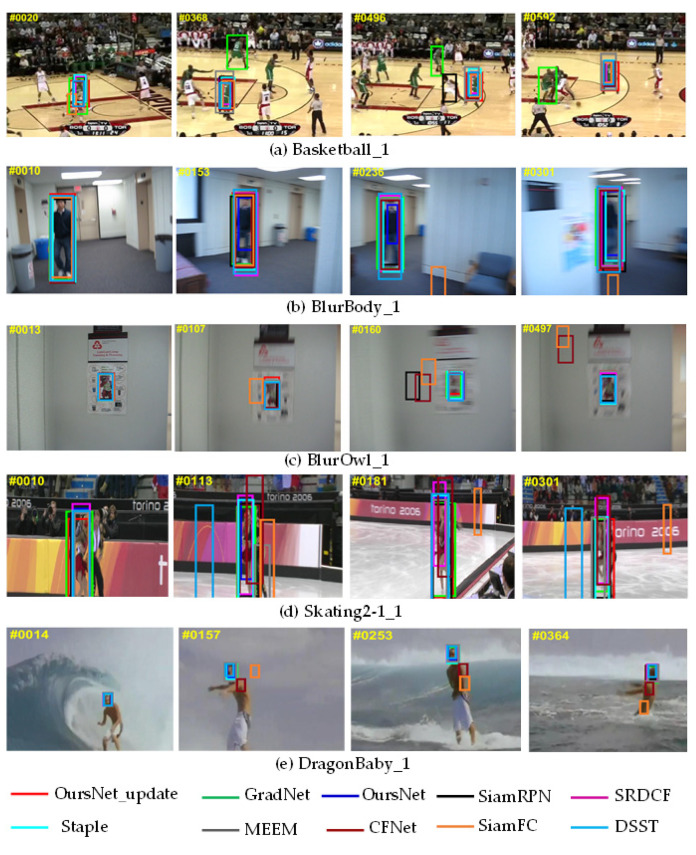
Sample tracking results of evaluated algorithms on different challenging sequences.

To further verify the model performance, the qualitative performance of the designed method and eight typical tracking algorithms were evaluated on the OTB benchmark, where the comparisons included SiamFC [3], SiamRPN [6], CFNet [32], GradNet [24], SRDCF [33], Staple [34], DSST [35], and MEEM [36]. The results of the visualization experiment in some moments of selected challenging test sequences are shown in Figure 10a–e.

As shown in Figure 10a, the video sequence basketball_1 has background similarity interference and background clutter, which can have some performance impact on the tracking process. In frame 20, the bounding box of the SiamFC tracker shifts slightly. From frames 368, 496, and 592, we found that the GradNet and SiamRPN trackers failed to track. The tracking algorithm in this paper benefits from the fact that the proposed feature-enhanced network can track the target better than other trackers. 

As observed in Figure 10b,c, in BlurBody_1 and BlurOwl_1 video sequences, the tracking algorithm in this paper fused the shallow and deep features of passing channel attention, while using the transformer to construct long-term dependencies. When there was target blurring, which led to the complete tracking failure of trackers such as SiamFC and CFNet, the tracker in this paper still demonstrated better robustness. 

As viewed in Figure 10d, there was fast-moving and similar interference in the Skating2-1_1 video sequence. SiamFC and Staple trackers could not track due to the lack of ability to extract feature representations. In contrast, the tracker proposed in this paper and other trackers could track the target robustly. 

As evidenced in Figure 10e, SiamFC and CFNet trackers failed to track the target accurately at 157 frames due to the interference factors of fast target change in the DragonBaby_1 video sequence. In this paper, we used a dynamic template updating mechanism, which could always accurately track the target position in the DragonBaby_1 video sequence where the target appearance changed or there was fast movement, reflecting the robustness of the tracking algorithm.

## 5. Conclusions

This paper proposes a tracking method combining feature enhancement and template update, which uses ResNet50 as a benchmark network for feature pre-extraction of template images and search images. The feature vectors obtained from it are fed into the proposed feature enhancement network, and the feature enhancement module consists of a channel attention mechanism and a reconfigured transformer. After passing the channel attention mechanism, the shallow features fuse with the deep features. Then, the internal relationships between features are established by encoders. A decoder is used to carry out the interaction of information with the output of the encoder. It enables the network to select the key information effectively and improve the feature representation.

Thanks to the introduction of a dynamic template update strategy that allows adaptive template updates when the appearance of the target changes and, simultaneously, enriches the temporal-context information between video frames, the robustness of the tracker is improved. The experimental comparison results show that the proposed tracking algorithm improved the tracking accuracy and success rate, and outperformed some existing classical trackers.

## Figures and Tables

**Table 1:** Performance results of SOTA tracker with an update mechanism on the LaSOT dataset.

Methods	AUC	$P_{Norm}$
OurNet_update	52.2	**60.5**
OurNet	51.6	60.0
TrTr-online	**55.1**	-
TrTr-offline	46.3	-
UpdateNet-DaSiamPRN	47.5	56.0
UpdateNet-SiamFC	34.9	43.7
DSiam	30.3	40.5
ECO	32.4	33.8

**Table 2:** Performance comparison on GOT-10k benchmark.

Methods	AO	$SR_{0.5}$	$SR_{0.75}$
OurNet-update	**55.8**	**66**	**42.7**
OurNet	55.2	66	40.8
ATOM	55.0	63.4	40.2
SiamRPN++	51.7	61.6	32.5
SPM	51.3	59.3	35.9
SiamRPN	46.3	54.9	25.3
THOR	44.7	53.8	20.4
SiamFCv2	37.4	40.4	14.4
SiamFC	34.8	35.3	9.8
GOTURN	34.7	37.5	12.4
ECO	31.6	30.9	11.1
MDNet	29.9	30.3	9.9
Staple	24.6	23.9	8.9
SRDCF	23.6	22.7	9.4

## Data Availability

Most data generated of analyzed during this study are included in the submitted article. The datasets involved in this paper are all public datasets.
